# Cost-Effectiveness Analysis of Efforts to Reduce Risk of Type 2 Diabetes and Cardiovascular Disease in Southwestern Pennsylvania, 2005-2007

**Published:** 2010-08-15

**Authors:** Kenneth J. Smith, Cindy L. Bryce, Heather E. Hsu, Mark S. Roberts, M. Kaye Kramer, Trevor J. Orchard, Gretchen A. Piatt, Miriam C. Seidel, Janice C. Zgibor

**Affiliations:** University of Pittsburgh, Pittsburgh, Pennsylvania; University of Pittsburgh School of Medicine; University of Pittsburgh, Pittsburgh, Pennsylvania; University of Pittsburgh, Pittsburgh, Pennsylvania; University of Pittsburgh, Pittsburgh, Pennsylvania; University of Pittsburgh, Pittsburgh, Pennsylvania; University of Pittsburgh, Pittsburgh, Pennsylvania; University of Pittsburgh, Pittsburgh, Pennsylvania; University of Pittsburgh, Pittsburgh, Pennsylvania

## Abstract

**Introduction:**

We assessed the cost-effectiveness of a community-based, modified Diabetes Prevention Program (DPP) designed to reduce risk factors for type 2 diabetes and cardiovascular disease.

**Methods:**

We developed a Markov decision model to compare costs and effectiveness of a modified DPP intervention with usual care during a 3-year period. Input parameters included costs and outcomes from 2 projects that implemented a community-based modified DPP for participants with metabolic syndrome, and from other sources. The model discounted future costs and benefits by 3% annually.

**Results:**

At 12 months, usual care reduced relative risk of metabolic syndrome by 12.1%. A modified DPP intervention reduced relative risk by 16.2% and yielded life expectancy gains of 0.01 quality-adjusted life-years (3.67 days) at an incremental cost of $34.50 ($3,420 per quality-adjusted life-year gained). In 1-way sensitivity analyses, results were sensitive to probabilities that risk factors would be reduced with or without a modified DPP and that patients would enroll in an intervention, undergo testing, and acquire diabetes with or without an intervention if they were risk-factor–positive. Results were also sensitive to utilities for risk-factor–positive patients. In probabilistic sensitivity analysis, the intervention cost less than $20,000 per quality-adjusted life-year gained in approximately 78% of model iterations.

**Conclusion:**

We consider the modified DPP delivered in community and primary care settings a sound investment.

## Introduction

Randomized controlled trials have demonstrated the efficacy of lifestyle interventions aimed at preventing or delaying onset of type 2 diabetes (1-4). The Diabetes Prevention Program (DPP) found that either medication or intensive lifestyle interventions could prevent progression from impaired glucose tolerance to diabetes (1). This randomized controlled trial provided study participants with individualized, resource-intensive management and oversight (5). During the 3 years of the DPP, diet and exercise reduced the risk that patients with impaired glucose tolerance would develop diabetes by 58% (1). These outcomes appear sustainable given follow-up data from the Da Qing Diabetes Prevention Study (6) and the Finnish Diabetes Prevention Study (7). Unfortunately, such programs can be expensive to implement and can exclude people with some comorbidities, who may benefit from even modest improvements in diet and physical activity.

Community-based lifestyle interventions adapted from the DPP (1) demonstrate effectiveness for improving the risk factors for diabetes in community settings (8-10), but their costs are largely unexplored. US cost-effectiveness studies of intensive lifestyle interventions to prevent diabetes have differed in their perspectives, time frames, and inclusion of prediabetes screening costs, producing cost-effectiveness ratios that range from $1,100 to $143,000 per quality-adjusted life-year (QALY) (11-13). However, intervention efficacy and cost data for these studies were largely based on the intensive strategies and resources used by the original DPP (14,15).

The Diabetes Prevention Support Center of the University of Pittsburgh Diabetes Institute developed a modified version of the DPP lifestyle intervention (mDPP) and tested its effectiveness in the community and local medical practice settings with patients at increased risk of diabetes or cardiovascular disease (CVD) (8,16). We assessed cost-effectiveness of this mDPP.

## Methods

### Study population

Diabetes Prevention Support Center faculty developed the Group Lifestyle Balance program by translating the original DPP lifestyle intervention (14) for distinct populations and measuring program effects in those populations. Intervention goals were to help patients with metabolic syndrome lose weight and improve at least 1 metabolic syndrome component. Investigators evaluated this intervention in different populations in 2 studies. In the first study, investigators assessed intervention effectiveness in 2 urban and 2 rural medical practices in southwestern Pennsylvania (16). They screened and recruited participants through the practices and hired trained preventionists who were health care professionals to deliver the program. The second study was a nonrandomized prospective trial to test intervention effectiveness in an urban, medically underserved community (8). The study held community-based screenings in targeted neighborhoods to identify and recruit eligible subjects, and a team of 2 health professionals and 2 lay health workers scheduled the lifestyle intervention sessions at worksites and churches.

Group Lifestyle Balance adapted the original DPP for use in group-based settings rather than individualized delivery and decreased the number of lessons from 16 to 12, offered for 12 to 14 weeks. Sessions were designed to achieve and maintain a 5% to 7% weight loss and to progressively raise activity levels to 150 minutes per week of moderately intense physical activity. Both studies assessed subjects for excess weight (body mass index [BMI] ≥25 kg/m^2^) and the following 4 components of metabolic syndrome, as defined by the National Cholesterol Education Program's Adult Treatment Panel III (17): waist circumference (>102 cm for men and >88 cm for women), high-density lipoprotein cholesterol (<40 mg/dL for men and <50 mg/dL for women), fasting glucose (≥100 mg/dL), and blood pressure (≥130/85 mm Hg). In addition, 1 study screened for triglyceride levels (≥150 mg/dL) (8).

### Development of a decision model

We used TreeAge Decision Pro Suite 2008 (TreeAge Software, Inc, Williamstown, Massachusetts) to construct a Markov decision model to estimate the incremental cost-effectiveness of a community-based mDPP. In the model, we used a base case that examined 55-year-old men and women at monthly intervals for 3 years. This time frame was chosen to limit projections regarding the continuing effectiveness of the mDPP, which is unknown; differing mDPP effectiveness assumptions over time were examined in sensitivity analyses. We defined the incremental cost-effectiveness as the additional cost of using an mDPP compared with providing usual care, divided by the additional clinical benefit of using the mDPP compared with providing usual care. For this model, usual care is the absence of a screening program and intervention.

In keeping with the reference case recommendations of the Panel on Cost-Effectiveness in Health and Medicine (18), we discounted future costs and benefits by 3% annually. We used a modified societal perspective in which the costs of patients' time were not included. To convert all monetary costs to the US dollar rate for 2000, we used the US Consumer Price Index. To account for changes in life expectancy and quality of life for diabetes-related health states, we used QALYs, which adjust for quality based on a utility weight, or preference, for the health state ranging from 0 (death, least preferred) to 1 (perfect health, most preferred).

Clinical outcomes and costs related to diabetes and complicated diabetes for both the mDPP and usual care were derived from the DPP (12,19), the Framingham Heart Study (20), and the United Kingdom Prospective Diabetes Study (12,21,22). Program costs, recruitment and retention rates, patient demographics, and program effectiveness were derived from the 2 community-based studies. mDPP costs were the costs of screening plus the personnel costs per patient  (Table).

### Basic model structure

**Figure 1:**
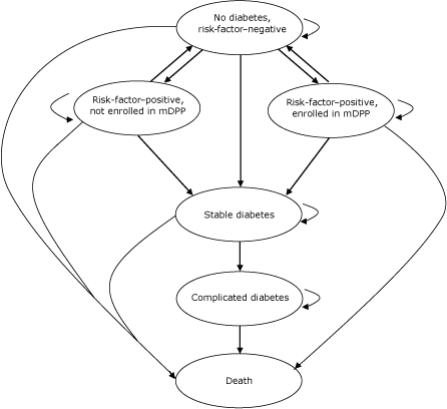
Model analyzing cost-effectiveness of a modified Diabetes Prevention Program (mDPP), southwestern Pennsylvania, 2005-2007. Ovals indicate health states. Subjects may remain in a health state (short curved arrow) or may move to a different health state (straight arrow or long curved arrow) during each model cycle.

To analyze the cost-effectiveness of an mDPP, we used the Markov model (Figure 1). At the start, subjects without a history of diabetes are evaluated once for risk factors for diabetes and CVD. Subjects are considered to be risk-factor–positive if they are overweight (BMI ≥25 kg/m^2^) and have at least 3 components of metabolic syndrome. They are also considered to be risk-factor–positive if they are overweight, have at least 2 components of metabolic syndrome, have a fasting glucose level of 100 mg/dL to 109 mg/dL, and have a physician referral to the intervention. They are considered to be risk-factor–negative if they do not meet either of these sets of criteria.

In the model, risk-factor–positive subjects are eligible for mDPP enrollment. Those enrolling in the program show metabolic syndrome resolution at rates found in the mDPP interventions during the first year of the model. Participants who begin the program but do not return for the 12-month follow-up are considered nonenrolled, thus accounting for withdrawal from the program. Those who do not enroll show a resolution of metabolic syndrome at the rate reported for the placebo arm of the DPP (19) during the 3 years of the model; this same rate of reduction is used for enrolled patients during model years 2 and 3 in the base case analysis. In sensitivity analyses, we also examined other assumptions for continued resolution of metabolic syndrome: 1) no further resolution after the first year for enrolled and nonenrolled patients and 2) no further resolution after the first year for enrolled patients but continued metabolic syndrome resolution at DPP placebo rates for all 3 model years in nonenrolled patients. Risk-factor–negative patients are ineligible for enrollment in the mDPP, and they develop metabolic syndrome at the rate reported for the placebo arm of the DPP (19).

Both risk-factor–positive and risk-factor–negative patients are at risk for developing diabetes at rates reported by the DPP. In patients who develop diabetes, the transition to complicated diabetes is preceded by a stable diabetes stage. Complications from diabetes include neuropathy, nephropathy, stroke, and coronary heart disease. In the model, patients in all health states can die; rates of death are based on age- and sex-specific US mortality (which accounts for baseline mortality) and the relative risks of death for metabolic syndrome, stable diabetes, and complicated diabetes (23).

### Sensitivity analyses

We performed 1-way sensitivity analyses and probabilistic sensitivity analyses on model input parameters. In these analyses, the parameters (Table) were varied either individually or collectively over their listed ranges, with 1,000 recalculations of incremental cost-effectiveness ratios based on random draws from the parameter distributions. Generally, without precise empirical data, sensitivity analyses rely on parameter distributions that reflect uncertainty and the range of likely values. In our analyses, cost data and utilities were varied over uniform distributions. Incidence and prevalence parameters were varied over beta distributions, relative risks were varied over log-normal distributions, and cost multipliers were varied over normal distributions.

The University of Pittsburgh institutional review board approved the protocols of both intervention studies and the cost-effectiveness analyses, and study participants provided informed consent.

## Results

By the 12-month point in the model, the mDPP intervention reduced metabolic syndrome risk at 1 year by 16.2% (compared with usual care, which reduced the risk of metabolic syndrome by 12.1%). During the 3-year time frame of the model, both costs and effectiveness of the mDPP were slightly higher than usual care. The mDPP costs totaled $2,528 (compared with $2,493 for usual care) and the effectiveness of the mDPP equaled 2.40 QALYs (compared with 2.39 QALYs for usual care). Taken together, the mDPP gained 0.01 QALYs (approximately 3.67 days) at an incremental cost of $34.50, equal to an incremental cost-effectiveness ratio of $3,420 per QALY. These results were due mainly to decreases in diabetes incidence with the mDPP. Without the mDPP, 9.6% of the cohort developed diabetes over 3 years; with the mDPP, 7.7% did. Over this period, little difference between groups was seen (1.1% vs 0.9%) in diabetes complication incidence, as broadly defined by the DPP.

**Figure 2:**
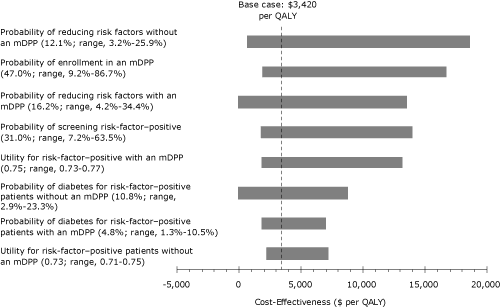
One-way sensitivity analyses assessing cost-effectiveness of a modified Diabetes Prevention Program (mDPP), southwestern Pennsylvania, 2005-2007. Horizontal bars depict the range of cost-effectiveness ratios for the values shown for each parameter. The vertical dotted line depicts the base case cost-effectiveness ratio. Variation of all other parameters not shown in the figure did not increase the cost-effectiveness ratio above $7,000 per quality-adjusted life-year (QALY) gained.

In 1-way sensitivity analyses, results were most sensitive to changes in risk-factor reduction with or without the mDPP, intervention rates, risk-factor–positive screening rates, and diabetes incidence rates in risk-factor–positive people with or without an intervention. Results were also sensitive to utilities for risk-factor–positive patients (Figure 2).

**Table:** 

Figure 2 shows the 1-way sensitivity analysis for 8 model parameters. For each parameter, we summarize the parameter values (baseline value; range: minimum, maximum) and provide the corresponding cost-effectiveness ratios (CERs).
For example, the first parameter listed is “Probability of reducing risk factors without an mDPP.” The baseline probability was 12.1%, but in sensitivity analyses, we varied this value from a low of 3.2% to a high of 25.9%. At the baseline value, the cost-effectiveness ratio was $3,420. If this probability decreases to 3.2%, then the cost-effectiveness ratio is $783 per QALY; if the probability increases to 25.9%, then the cost-effectiveness ratio is $18,580. We summarize this information as follows:
Probability of reducing risk factors without an mDPP: 12.1%; range, 3.2%-25.9% ($3,420; range, $783-$18,580)
Analogous summaries for the remaining 7 parameters are
Probability of enrollment in an mDPP: 47.0%; range, 9.2%-86.7% ($3,420; range, $16,707-$1,911) Probability of reducing risk factors with an mDPP: 16.2%; range, 4.2%-34.4% ($3,420; range, $13,087-$0) Probability of screening risk-factor–positive: 31.0%; range, 7.2%-63.5% ($3,420; range, $14,046-$1,818) Utility for risk-factor–positive patients with an mDPP: 0.75; range, 0.73-0.77 ($3,420; range, $13,178-$1,926) Probability of diabetes for risk-factor–positive patients without an mDPP: 10.8%; range, 2.9%-23.3% ($3,420; range, 8,505-$0) Probability of diabetes for risk-factor–positive patients with an mDPP: 4.8%; range, 1.3%-10.5% ($3,420; range, $7,085-$1,911) Utility for risk-factor–positive patients without an mDPP: 0.73; range, 0.71-0.75 ($3,420; range, $2,280-$7,301)

**Table:** 

**Model Parameter**	**Parameter Value**			**Cost-Effectiveness, $ per QALY**		

	**Base case**	**Min value**	**Max value**	**Base case**	**Low CER**	**High CER**
Probability of reducing risk factors without an mDPP	12.1%	3.2%	25.9%	3,420	783	18,580
Probability of enrollment in an mDPP	47.0%	9.2%	86.7%	3,420	1,911	16,707
Probability of reducing risk factors with an mDPP	16.2%	4.2%	34.4%	3,420	0	13,087
Probability of screening risk-factor–positive	31.0%	7.2%	63.5%	3,420	1,818	14,046
Utility for risk-factor–positive patients with an mDPP	0.75	0.73	0.77	3,420	1,926	13,178
Probability of diabetes for risk-factor–positive patients without an mDPP	10.8%	2.9%	23.3%	3,420	0	8,505
Probability of diabetes for risk-factor–positive patients with an mDPP	4.8%	1.3%	10.5%	3,420	1,911	7,085
Utility for risk-factor–positive patients without an mDPP	0.73	0.71	0.75	3,420	2,280	7,301

Abbreviations: QALY, quality-adjusted life-year; Min, minimum; Max, maximum; CER, cost-effectiveness ratios; mDPP, modified Diabetes Prevention Program.

When base case values were used for all parameters, an mDPP intervention cost $3,420 per QALY. When parameters were varied to the extremes of the ranges shown in the Table, the cost-effectiveness ratio remained less than $20,000 per QALY. The cost-effectiveness ratio rose highest, $18,600 per QALY, when the probability of reducing risk factors in the absence of an mDPP intervention was increased from 12.1% in the base case to 25.9% (Figure 2). In addition, the cost-effectiveness of the mDPP intervention remained less than $17,000 per QALY when the proportion of patients who screen positive for risk factors increased to 63.5% (base 31%), when the proportion of risk-factor–positive patients who enroll in the intervention increased to 86.7% (base 47.0%), when the probability of risk-factor reduction increased to 34.4% (base 16.2%), and when the relative risk of diabetes given risk factor positivity with or without an mDPP decreased by two-thirds. When all other parameters listed in the Table were varied in 1-way sensitivity analyses across their specified ranges, the cost-effectiveness ratio did not exceed $7,000 per QALY. Thus, results favoring the mDPP were stable to variations in risk-factor reduction and diabetes risks that might be seen in differing populations. Varying the discount rate for costs and effectiveness (base 3%) from 0% to 5% changed model results by less than $400 per QALY gained because of the brief 3-year span of the model.

In a separate sensitivity analysis examining differing assumptions for metabolic syndrome reduction, if we assume no reduction in metabolic syndrome for enrolled or nonenrolled patients after model year 1, the cost-effectiveness ratio of the intervention increased slightly, to $3,400 per QALY gained. If enrolled patients have no further reduction in metabolic syndrome after the first year but nonenrolled patients continue metabolic syndrome reduction through the 3 years at rates seen in the DPP placebo arm, the intervention would cost $7,270 per QALY.

**Figure 3:**
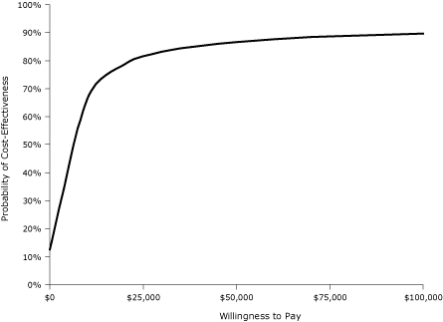
Probabilistic (Monte Carlo) sensitivity analyses assessing cost-effectiveness of a modified Diabetes Prevention Program (mDPP), southwestern Pennsylvania, 2005-2007. The acceptability curve depicts the likelihood of an mDPP lifestyle intervention being favored for a given cost-effectiveness ceiling threshold (willingness to pay).

In a probabilistic sensitivity analysis, when all parameters were varied simultaneously across their ranges, the mDPP intervention cost less than $20,000 per QALY gained in approximately 78% of model iterations and less than $50,000 per QALY in approximately 86% of iterations (Figure 3). This analysis assumed independence among parameter values and did not account for covariance between parameters, which tends to broaden result ranges. Thus, we may have underestimated the probabilities.

**Table:** 

**Willingness to Pay, $**	**Probability of Cost-Effectiveness, %**
0	12
5,000	50
10,000	67
15,000	75
20,000	79
25,000	82
30,000	84
35,000	85
40,000	86
45,000	87
50,000	87
55,000	87
60,000	88
65,000	88
70,000	88
75,000	88
80,000	89
85,000	89
90,000	89
95,000	89
100,000	89

When the utility weight for risk-factor–positive patients not enrolled in an intervention (ie, receiving usual care) was set equal to the utility weight for patients enrolled in an mDPP, the mDPP intervention cost $8,300 per QALY and yielded life expectancy gains of 0.004 QALYs (approximately 1.63 days) for enrollees. We also examined scenarios using sets of parameter values unfavorable to the mDPP intervention. If we simultaneously assume no utility differences with risk factors between intervention groups (as above), a 9.8% diabetes risk on the mDPP (so that the risk is only slightly better than the 10.8% risk of no mDPP), and a utility of 0.77 with no risk factors (instead of a low value of 0.84), then the mDPP cost $56,200 per QALY gained. If we add to this scenario a decrease in mDPP-related benefit (from 16.2% to 14.1%, a 2 percentage point difference compared with no mDPP), then the mDPP cost $95,400 per QALY.

## Discussion

The mDPP was designed to teach groups of people how to change their diet and lifestyle to reduce their risk for diabetes and CVD. When we examined the costs and effects of implementing the mDPP intervention in a community setting, we found that at 12-month follow-up the mDPP reduced the relative risk of metabolic syndrome by 16.2% and yielded a life-expectancy gain of 3.67 days at a cost of $3,420 per QALY gained. Even when we varied parameter values in sensitivity analyses, the cost-effectiveness ratio remained less than $20,000 per QALY. Ratios less than $20,000 are generally considered to provide strong evidence in favor of adopting an intervention (31).

In analyzing the mDPP, we used several conservative practices and assumptions that would be expected to negatively bias our findings. First, we used outcome parameters based on data from real-world mDPPs in which participation ranged from attending 1 session to attending 12 sessions (mean, 8.9 sessions; median, 10 sessions). In contrast to usual study designs, in which attrition and dropout rates are final states (ie, patients do not return after dropping out), our study design included patients who skipped sessions throughout the 12-week course. Second, our design was based on an intent-to-treat analysis in which all patients who attended at least 1 session were included and any patient who did not return for the 12-month follow-up was assumed to be risk-factor–positive even if the 3-month data indicated resolution in the patient's metabolic syndrome. Third, we modeled the effects of the intervention over a 3-year time frame. If the effects are actually sustained beyond 3 years, as other studies suggest (6,12), then our modeling approach underestimates the cost-effectiveness of the intervention. Given the short time frame of our analysis, the factors that contributed the most to intervention effectiveness were the changes in quality of life that are due to avoidance of diabetes and its complications. Over longer time frames, other factors could have a greater impact.

Our study has 2 limitations that deserve mention. First, we used costs in 2000 US dollars. This decision might underestimate the costs of providing the mDPP intervention and usual care today, but it would not be expected to greatly underestimate the costs of the mDPP intervention relative to the costs of usual care in any particular year. Moreover, the choice of base year would not be expected to influence the criteria for cost-effectiveness (31). Second, as with any modeling exercise, we imposed several simplifications. For example, although the probabilities of acquiring diabetes, progressing to complicated diabetes, and dying of diabetes or other causes depend on a large number of covariates, we included only the most common covariates. Addressing these limitations would require the performance of a large-scale trial to compare the mDPP intervention with usual care for a long follow-up period. In the absence of such a trial, we believe that using the control arm of the original DPP as the comparison cohort for the mDPP intervention was a reasonable alternative.

Both the original DPP and the mDPP provide study participants with instructions about diet and physical activity. The main difference is that the DPP provides individualized instruction to participants with specific types of comorbidities, whereas the mDPP provides group instruction and can be applied to participants with a larger range of comorbidities. Although the DPP was found to be economically reasonable (12), it is more expensive than the mDPP and its complexity and rigor make it more difficult for medical practitioners to fully implement. The mDPP is simpler and less time-consuming to implement. When delivered in community and primary care settings, it is cost-effective and appears to be a sound investment.

## Figures and Tables

**Table.** Base Case Values for Decision Model and Ranges Examined in Sensitivity Analyses of Efforts to Reduce Risk of Type 2 Diabetes and Cardiovascular Disease, Southwestern Pennsylvania, 2005-2007

**Parameter**	Base Case Value	Type of Distribution	Range Examined	Reference
**Cohort characteristics**				
Starting age, y	55	Uniform	45–65	Assumption
Women, %	75	Beta	48.2-94.0	mDPP data (8,16)
African American, %	27.1	Beta	6.5-55.8	mDPP data (8,16)
Angina, %	3.8	Beta	1.0-8.3	mDPP data (8,16)
Hypertension, treated, %	84.9	Beta	4.5-100	mDPP data (8,16)
History of cardiac arrest or MI, %	1.9	Beta	0.5-4.2	mDPP data (8,16)
History of stroke, %	1.9	Beta	0.5-4.2	mDPP data (8,16)
Peripheral vascular disease, %	4.7	Beta	1.3-10.2	mDPP data (8,16)
**Probabilities, %**				
Probability of screening risk-factor–positive	31.0	Beta	7.2-63.5	mDPP data (8,16)
Probability of enrollment	47.0	Beta	9.2-86.7	mDPP data (8,16)
**Yearly probability of acquiring diabetes, %**				
Not in prevention program, risk-factor–positive	10.8	Beta	2.9-23.3	Herman et al (12)
Not in prevention program, risk-factor–negative	0.4	Beta	0.05-0.75	Fox et al (20)
In prevention program	4.8	Beta	1.3-10.5	Herman et al (12)
Yearly probability of becoming risk-factor–positive	4.0	Beta	1.0-8.7	Orchard et al (19)
Yearly probability of progressing to complicated diabetes	7.5	Beta	2.0-16.3	Herman et al (12), Kothari et al (21), Wilson et al (22)
**Yearly probability of reducing risk factors, %**				
Not in prevention program	12.1	Beta	3.2-25.9	Orchard et al (19)
In prevention program	16.2	Beta	4.2-34.4	mDPP data (8,16)
**Relative risk of death**				
Risk-factor–positive	1.7	Log-normal	1.5-1.8	Lakka et al (23)
Risk-factor–negative	1.0	NA	Not varied	Assumption
Stable diabetes	2.0	Log-normal	1.8-2.2	Moss et al (24)
Complicated diabetes	2.4	Log-normal	2.2-2.6	Fuller et al (25)
**Utilities**				
No diabetes, risk-factor–positive, not in prevention program	0.73	Uniform	0.71-0.75	Herman et al (12), Coffey et al (26)
No diabetes, risk-factor–positive, in prevention program	0.75	Uniform	0.73-0.77	Herman et al (12), Coffey et al (26)
No diabetes, risk-factor–negative	0.88	Uniform	0.84-0.92	Gold et al (27)
Stable diabetes	0.69	Uniform	0.66-0.72	Herman et al (12), Coffey et al (26), Zhou et al (28)
Complicated diabetes	0.59	Uniform	0.51-0.68	Herman et al (12), Coffey et al (26), Zhou et al (28)
**Costs and multipliers**				
Screening, risk-factor–positive, $	35	Uniform	18-53	mDPP data (8,16)
Screening, risk-factor–negative, $	32	Uniform	16-48	mDPP data (8,16)
Prevention program, $	219	Uniform	110-329	mDPP data (8,16)
Risk-factor–positive (yearly), $	1,296	NA	Not varied	Herman et al (12)
Multiplier for female	1.14	Normal	1.05-1.25	Herman et al (12)
Multiplier for African American	0.82	Normal	0.70-0.95	Herman et al (12)
Risk-factor–negative (yearly), $	616	NA	Not varied	MEPS^a^
Base diabetes cost (yearly), $	1,684	NA	Not varied	Herman et al (12)
Multiplier for female	1.25	Normal	1.14-1.35	Herman et al (12)
Multiplier for African American	0.82	Normal	0.70-0.95	Herman et al (12)
Base complicated diabetes cost (yearly), $	1,684	NA	Not varied	Herman et al (12)
Multiplier for female	1.25	Normal	1.14-1.35	Herman et al (12)
Multiplier for African American	0.82	Normal	0.70-0.95	Herman et al (12)
Multiplier for angina	1.73	Normal	1.31-2.14	Herman et al (12)
Multiplier for hypertension, treated	1.24	Normal	1.10-1.37	Herman et al (12)
Multiplier for history of cardiac arrest or MI	1.90	Normal	1.64-2.17	Herman et al (12)
Multiplier for history of stroke	1.30	Normal	1.20-1.40	Herman et al (12)
Multiplier for peripheral vascular disease	1.31	Normal	1.10-1.53	Herman et al (12)

Abbreviations: mDPP, modified Diabetes Prevention Program; MI, myocardial infarction; NA, not applicable; MEPS, Medical Expenditure Panel Survey.

a The yearly cost for risk-factor–negative ($616) was computed using the subset of MEPS respondents who had incurred health care expenses during the year and who reported a perceived health status of good, very good, or excellent (29).
